# Influence of Selected Abiotic Factors on Triterpenoid Biosynthesis and Saponin Secretion in Marigold (*Calendula officinalis* L.) in Vitro Hairy Root Cultures

**DOI:** 10.3390/molecules24162907

**Published:** 2019-08-10

**Authors:** Abdulwadood Shakir Mahmood Alsoufi, Cezary Pączkowski, Marek Długosz, Anna Szakiel

**Affiliations:** 1 Department of Biology, College of Science, University of Tikrit, P.O. Box 42, Tikrit 34001, Iraq; 2 Department of Plant Biochemistry, Faculty of Biology, University of Warsaw, 1 Miecznikowa Street, 02-096 Warsaw, Poland

**Keywords:** *Calendula officinalis*, elicitation, hairy roots, heavy metals, sterols, triterpenoids, ultrasound, UV-radiation

## Abstract

The aim of the study was the evaluation of the efficiency of selected abiotic elicitors, i.e., silver and cadmium ions, ultrasound, and UV-C irradiation, in the stimulation of triterpenoid biosynthesis, accumulation, and saponin secretion in *Calendula officinalis* hairy root cultures. Apart from the possible enhancement of triterpenoid production, the relationship between primary and secondary metabolism (represented respectively by sterols and pentacyclic triterpenes), modifications of the sterol compositional profile, and fluctuations in the total triterpenoid content were monitored in the performed experiments. The main phenomenon observed as a response to heavy metal treatment was the stimulation (up to 12-fold) of the secretion of saponins, accompanied by significant changes in sterol composition. Ultrasound stimulated the secretion of saponins (up to 11-fold); however, it exerted diverse influences on the triterpenoid content in hairy root tissue (stimulating or decreasing) depending on the duration of the exposure to the elicitor. UV-C radiation caused a slight increase in the content of both sterols and saponins in hairy root tissue, and stimulated saponin secretion up to 8.5-fold. The expected symptoms of the competition between the biosynthetic pathways of sterols and pentacyclic triterpenoids were less evident in reactions to abiotic stressors than those reported previously for biotic elicitors.

## 1. Introduction

Triterpenoids represent a large group of plant isoprenoids synthesized from the C30 precursor squalene, a linear hydrocarbon, which is oxidized to 2,3-oxidosqualene and then rearranged by special enzymes, oxidosqualene cyclases, to various cyclic structures [1,2]. Two main groups can be distinguished among these derived structures: steroids, i.e., tetracyclic compounds based on the perhydro-1,2-cyclopentano-phenanthrene moiety, and pentacyclic triterpenoids possessing a 5-ring carbon skeleton in various arrangements. Steroids contain a characteristic class of compounds with a hydroxyl group at C-3 called sterols. Plant sterols can exist in plants in a basic free form, but also as esters with fatty or phenolic acids, or as glycosides and acylated steryl glycosides [3,4]. Pentacyclic triterpenoids can be classified according to their type of skeleton into oleananes, ursanes, lupanes, taraxasteranes, etc. They can exist in plants in free or bound forms, i.e., as esters or glycosides, referred to as saponins. Steroids and pentacyclic triterpenoids distinctly differ in the functions they perform in plants, and therefore they are commonly regarded as primary and secondary (or more aptly: “specialized”) metabolites, respectively. Sterols are constituents of plant membranes, and they participate in the regulation of their fluidity and permeability. They also serve as precursors of brassinosteroid hormones, whereas pentacyclic saponins are believed to play an important role in plant chemical defense and interactions with the environment. Therefore, the step of 2,3-oxidosqualene cyclization is often regarded as a branch point between primary and secondary triterpenoid metabolism [1].

Many isoprenoid compounds, including some triterpenoids, have significant commercial value as drugs, nutrients, pigments, fragrances, polymers, and agrochemicals. Their extraction from native plants often leads to low yields, and chemical synthesis is usually not viable due to the structural complexity of these compounds. Attempts have been made to solve these problems by metabolic and genetic engineering in plants, cell-based and cell-free “synthetic systems” with the use of plant or microbial enzymes, as well as by the application of various in vitro cultures [1,5,6]. Triterpenoid concentrations in plant cells and tissue cultures are generally lower than those in intact plants. Only a few examples of plant in vitro cultures producing more triterpenoids than the native plant have been reported so far, e.g., calluses from *Actinidia arguta* synthesized 20-times more oleanolic and ursolic acids than the corresponding plant, and *Camptotheca acuminata* calluses produced higher amounts of polyhydroxylated oleanolic and ursolic acid derivatives than either the leaves or roots of the intact plant [7]. Other plant cultures have to be stimulated for triterpenoid production, and the most commonly used strategy is the empirical application of various elicitors.

Due to its practical feasibility and effectiveness, elicitation is one of the most commonly applied strategies for enhancing the production of desired compounds (particularly secondary metabolites) in plant biotechnology. For intact plants, elicitation can be considered as a defense reaction to stress conditions or external factors (referred to as “elicitors”), usually resulting in a process of inducing or enhancing the synthesis of plant defense metabolites to ensure the plant’s survival, persistence, and competitiveness [8]. In biotechnology, elicitation is often defined simply as the induced or enhanced biosynthesis of metabolites due to the addition of trace amounts of elicitors [9]. Elicitors can be divided into physical or chemical, exogenous or endogenous (compounds released from plants after infection by pathogen), and specific or general. However, the most convenient and widely accepted classification is the split into two categories: abiotic and biotic [10,11]. Abiotic elicitors can be categorized as either chemical substances of non-biological origin, e.g., mineral salts, heavy metals, or physical factors/conditions, such as light (UV-B, UV-C radiation), temperature (heat or cold), ultrasound, and osmotic stress. Their effect depends on many parameters, such as the concentration of elicitor or the intensity of its action, the growth stage of the culture, the period of contact, and the time course of the elicitation. Although abiotic effectors have been less extensively exploited than biotic elicitors, they should not be regarded as less important. A number of interesting reports showing the efficiency of abiotic elicitors for the enhancement of the production of phytochemicals in in vitro cultures have been published in the last decade [9].

Due to a wide range of biological properties and pharmacological applications, there is significant demand for triterpenoid saponins, particularly those applied in phytomedicines and as immunoadjuvants in vaccines. Therefore, various strategies of elicitation were designed for increasing the biosynthesis of saponins, e.g., ginsenosides from *Panax ginseng* or centellosides from *Centella asiatica*, in cell cultures as well as in hairy or adventitious root cultures. Both biotic (jasmonic acid and its methyl ester, salicylic acid, auxins, cytokinins, gibberellins, yeast extract, oligosaccharides) and abiotic (osmotic stress, fluorescent light, temperature, copper ions, additional oxygen supply) elicitors were tested, exerting positive or negative effects on saponin production [12].

Recently, we reported the influence of selected biotic elicitors, jasmonic acid and chitosan, on the biosynthesis, accumulation, and secretion of triterpenoids in two lines of *Calendula officinalis* in vitro hairy root culture, obtained as a result of transformation with the wild type *Agrobacterium rhizogenes* strain ATCC 15834 [13]. *C. officinalis* hairy roots produce triterpenoid saponins (oleanolic acid glycosides) in varied yields and excrete these compounds into the culture medium [14,15]. Jasmonic acid was found to be a very effective elicitor, increasing both the accumulation of oleanolic acid saponins in the hairy root tissue and their secretion to the medium with a parallel decrease in the biosynthesis and accumulation of sterols. Chitosan slightly increased the accumulation and secretion of oleanolic acid saponins and increased the total content of sterols [13]. In the present work, we studied the effect of selected abiotic factors, i.e., silver and cadmium ions, ultrasound, and UV-C irradiation, on triterpenoid productivity in two *C. officinalis* hairy root lines differing in origin from initial explant. Silver and cadmium ions are included within the trace elements considered as heavy metals. Besides their evident toxicity for the native plants, these ions can be applied as efficient abiotic elicitors in in vitro cultures [9]. Ultrasound, a longitudinal pressure wave (often regarded as a “mechanical wave”), has been applied to improve the production of valuable secondary metabolites in plant cell cultures of numerous plant species, as well as to stimulate the release of these compounds to the surrounding medium [16]. Ultraviolet radiation (UV) has also been used as an abiotic factor stimulating the biosynthesis of various secondary metabolites in plant in vitro cultures [9,17]. Furthermore, such abiotic elicitors as ultrasound or UV have an additional advantage of being extremely low-cost and technically feasible, avoiding the need to supply external chemical substances and with a decreased risk of microbial contamination. Thus, abiotic elicitation can become one of the future strategies for sustainable production of saponins at industrial scale.

Apart from the evaluation of the possible enhancement of triterpenoid production in *C. officinalis* hairy roots with the use of abiotic elicitors, the aim of the present study was to investigate the relationship between primary and secondary metabolism (represented respectively by sterols and pentacyclic triterpenoids) triggered by stress conditions. The accumulated knowledge on triterpenoids occurring in *C. officinalis* plant, suggests that obtained in vitro cultures can serve as a research model for monitoring the modifications of compositional profile or fluctuations in triterpenoid content. Thus, the obtained results can contribute to understanding the participation of the two group of compounds, sterols, and pentacyclic triterpenoids, in a response and adaptation to various types of environmental stress.

## 2. Results and Discussion

### 2.1. Determination of Triterpenoids in Hairy Roots and Culture Medium

**Figure 1:**
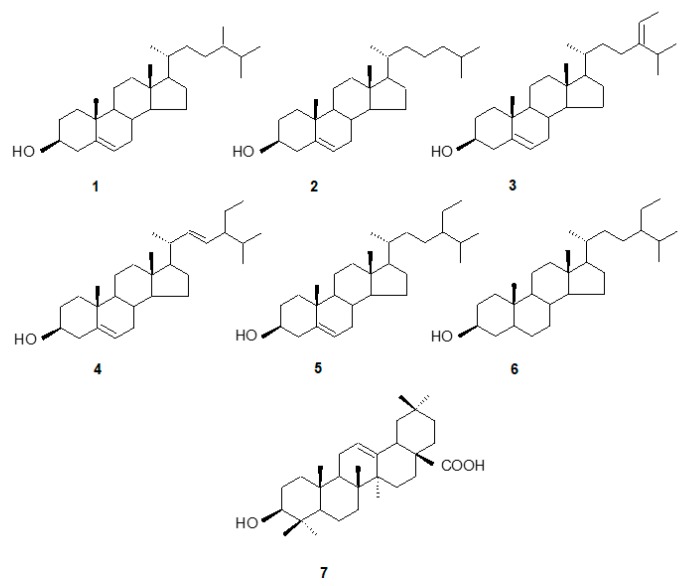
The structures of compounds identified in *Calendula officinalis* hairy root cultures (CC16 and CH2 lines). (**1**) campesterol, (**2**) cholesterol, (**3**) isofucosterol, (**4**) stigmasterol, (**5**) sitosterol, (**6**) sitostanol, (**7**) oleanolic acid.

The main sterols identified by GC-MS in *C. officinalis* hairy roots were: campesterol [(24*R*)-ergost-5-en-3β-ol], cholesterol [cholest-5-en-3β-ol], isofucosterol [stigmasta-5,24(28)-dien- 3-ol], stigmasterol [(22*E*)-stigmasta-5,22-dien-3β-ol], sitosterol [stigmast-5-en-3β-ol], and its fully hydrogenated derivative, sitostanol [stigmastan-3β-ol, synonym: stigmastanol] (Figure 1). The compounds were identified by their chromatographic mobility, mass spectra, and retention times, and quantified by an external standard method, as described in chapter 3.5. As reported in our previous papers [13,18], different *C. officinalis* hairy root lines can vary slightly in their sterol profile of (particularly with regard to the presence or absence of isofucosterol) and can accumulate intermediates of their biosynthetic pathways (e.g., cycloartanol and 24-methylenecycloartanol). However, in all basic, non-elicited lines, the predominant compounds remain the same, i.e., stigmasterol as the principal compound followed by sitosterol, as detected in natural *C. officinalis* roots [18]. Nevertheless, some alterations in the ratio among individual compounds were observed after the elicitation of hairy roots with jasmonic acid and chitosan [13], and thus such phenomena can be expected after treatment with other elicitors and different stress factors.

Apart from sterols, the fraction of neutral triterpenoids may contain other compounds in a free form, e.g., pentacyclic alcohols, aldehydes, and ketones, as well as their methoxy ethers and acetate esters, which cofractionate with free neutral triterpenoids due to a similar chromatographic mobility. The presence of small amounts of pentacyclic triterpenoids (α-amyrenone, α-amyrin, lupeol, oleanolic acid methyl ester, and 3-acetyloxy oleanolic acid methyl ester) was reported previously in another line of *Calendula* hairy roots [18]; however, in the two lines applied in the present study their occurrence was not confirmed. In the hydrolyzed ester fractions, only trace amounts of sterols were detected, and therefore they were not quantified and considered in the results presented in this study.

Oleanolic acid is the aglycone of *Calendula* saponins, and it occurs both in the native plant and in in vitro cultures mainly in this form. Only small amounts of free, non-glycosylated oleanolic acid had been detected in *Calendula* native plants [19]. In the present study, free oleanolic acid was not detected in measurable amounts in the fraction of triterpenoid acids; it was also not detected in any of the ester fractions obtained after alkaline hydrolysis. In turn, as expected, oleanolic acid was detected as an aglycon of saponins, which were either accumulated in hairy root tissue or released to the culture medium. It was analyzed quantitatively after acid hydrolysis of methanolic extracts of the hairy roots and butanolic extracts of the culture medium, respectively. The identity of the oleanolic acid present in the *Calendula* hairy roots and the culture medium was confirmed by GC-MS, and its quantification was carried out by GC as described in chapter 3.4.

### 2.2. Elicitation of Calendula Hairy Roots with Abiotic Elicitors

#### 2.2.1. Effect of Heavy Metals (Silver and Cadmium)

Elicitation of *C. officinalis* hairy roots with Ag^+^ and Cd^2+^ ions (supplied as silver nitrate AgNO_3_, and cadmium chloride CdCl_2_) was performed according to the procedure described in chapter 3.2.1.

The CC16 line was treated by four concentrations of silver nitrate and cadmium chloride: 25, 50, 100, and 150 μM. All concentrations significantly reduced both the fresh mass and dry weight of the roots (by 47–77% in accordance with increasing concentrations, see Figures S1 and S2). The highest concentration (150 μM) of both salts exerted a visibly harmful effect on the hairy roots, which became very dark and stopped growing. Therefore, the CH2 line was treated only by concentrations of 25, 50, and 100 μM. The CH2 line seemed to be less sensitive to the influence of Cd^2+^ ions, at least at the lowest applied concentration (25 µM), where no decrease in the fresh or dry mass was noted. However, at higher concentrations, particularly 100 µM, the decrease in both fresh and dry mass reached 50–66%. It should be emphasized that even low concentrations of heavy metals led to significant changes in the root morphology. The roots became darker, thicker, and significantly less branched, particularly after Ag^+^ treatment (Figure S3).

Elicitation with the tested heavy metal ions exerted diverse effects on the accumulation of oleanolic acid glycosides in the tissue of the two hairy root lines.

**Figure 2:**
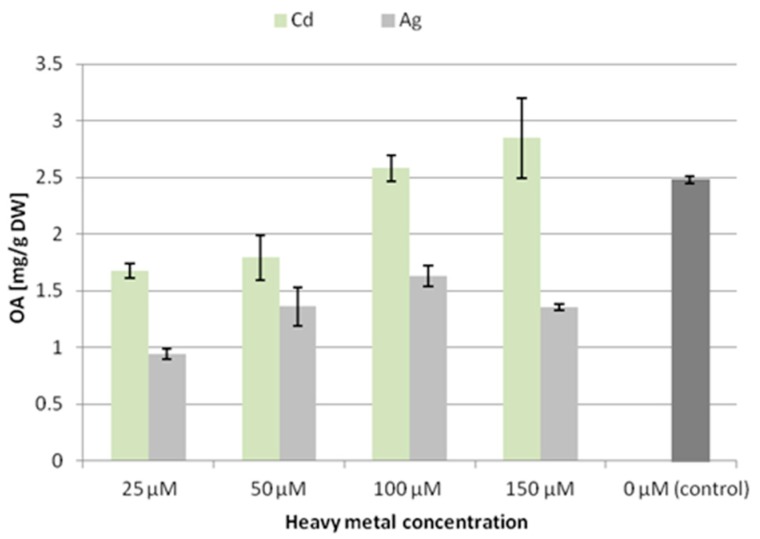
The content of oleanolic acid in hairy root tissue (CC16 line) after elicitation with silver and cadmium ions. OA—oleanolic acid.

In the CC16 line, the content of oleanolic acid (released from hydrolyzed saponins) decreased significantly at all the applied concentrations of silver ions, with the lowest level (decreased by 62% compared to the control) after treatment with 25 µM Ag^+^. In contrast, in samples treated with cadmium ions, the content of oleanolic acid decreased only at the two low concentrations, whereas it increased by 15% after treatment with 150 µM Cd^2+^ (Figure 2).

**Figure 3:**
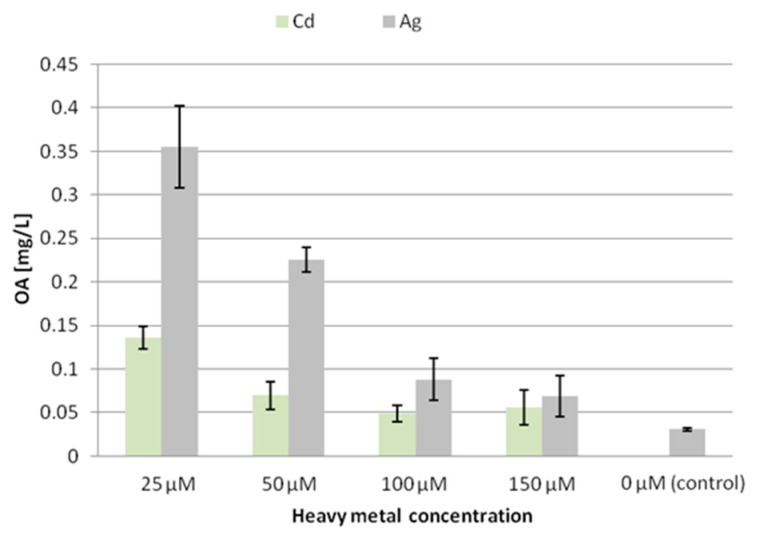
The content of oleanolic acid in the medium (CC16) after elicitation with silver and cadmium ions. OA—oleanolic acid.

The secretion of oleanolic acid glycosides from the CC16 line hairy roots increased after treatment with both tested heavy metals, although much more significantly after elicitation with silver ions (Figure 3). The strongest effect was observed at a concentration of 25 μM, where the amount of oleanolic acid secreted to the culture medium increased 5-fold and 12-fold after treatment with Cd^2+^ or Ag^+^, respectively. In turn, Cd^2+^ ions at concentrations of 100 and 150 μM did not show statistically significant increases in oleanolic acid in the culture medium. The lowest secretion of oleanolic acid saponins after elicitation with Ag^+^ ions (however, still more than 2-fold) was observed at a concentration 150 μM. Thus, the influence of silver ions on the secretion of oleanolic acid glycosides in the line CC16 was found to be inversely concentration-dependent.

The strong stimulation of the secretion of oleanolic acid glycosides exerted by the lower concentrations of both applied heavy metal ions is correlated with the lowest levels of these compounds accumulated in hairy root tissue. Therefore, the decrease in the content of oleanolic acid in the tissue can be explained by their increased secretion to the surrounding medium. At higher concentrations of heavy metal ions, the secretion of oleanolic acid glycosides was not as intensive, and thus a greater amount of these compounds was accumulated in the tissue. This correlation is particularly remarkable in the case of cadmium ions.

**Figure 4:**
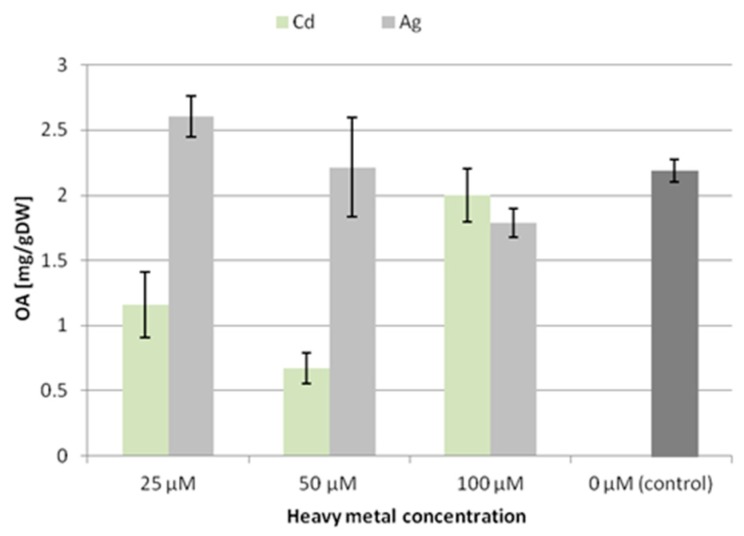
The content of oleanolic acid in hairy root tissue (CH2 line) after elicitation with silver and cadmium ions. OA—oleanolic acid.

**Figure 5:**
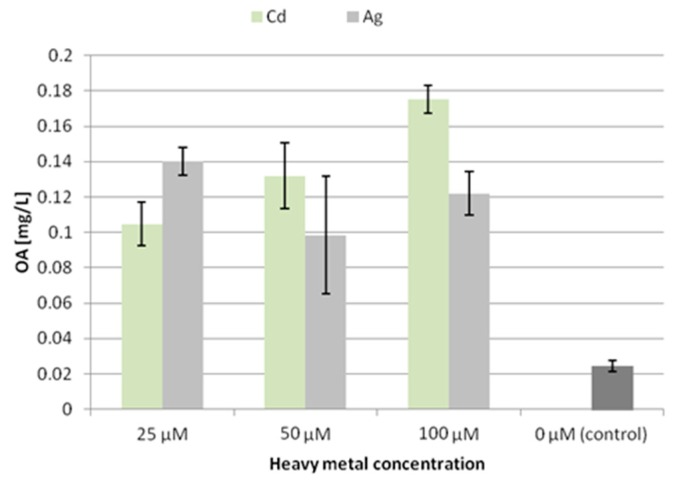
The content of oleanolic acid in the medium (CH2 line) after elicitation with silver and cadmium ions. OA—oleanolic acid.

The trends of accumulation and secretion of oleanolic acid glycosides after treatment with heavy metals noted in the CH2 line (Figure 4 and Figure 5) differ in some aspects from those observed for the CC16 line.

After treatment with silver ions, the content of saponins accumulated in the CH2 line root tissue increased at the lowest applied concentration (25 μM Ag^+^) by 14% and decreased by 18% at 100 μM Ag^+^. In turn, after treatment with Cd^2+^ ions, the content of saponins accumulated in the root tissue decreased, particularly at the two lower concentrations, i.e., almost by 50% at both 25 and 50 μM Cd^2+^. Thus, the accumulation of saponins in the CH2 line hairy roots was affected more strongly by Cd^2+^ ions than Ag^+^ ions, and these effects were opposite to those observed for the CC16 line. It can be concluded that the two lines of hairy roots, CC16 and CH2, significantly differ in their sensitivity to the two applied metal ions regarding the effect exerted on their accumulation of oleanolic acid glycosides.

Elicitation with Ag^+^ or Cd^2+^ ions increased the secretion of oleanolic acid glycosides in the CH2 line at all the applied concentrations, but with opposite tendencies. The saponin secretion was increased 4.5-fold by comparison to the control at 25 μM Cd^2+^, and almost 8-fold at 100 μM Cd^2+^. Conversely, the secretion increased almost 5.5-fold after treatment with 25 μM Ag^+^, and only 4-fold at 100 μM Ag^+^. Thus, the enhancement of secretion of oleanolic acid glycosides in the CH2 line was directly proportional to growing cadmium ion concentration, whereas it was inversely proportional to silver ion concentration.

A similar inverse relationship between saponin secretion and silver ion concentration was also observed for the CC16 line. Thus, the influence of the tested heavy metal ions on saponin secretion in the two hairy root lines differed, particularly with respect to the cadmium ion concentration.

The content of sterols in the CC16 line after elicitation with silver ions decreased by almost 50% compared to the control at a concentration of 25 µM Ag^+^ (Table 1). According to the results described above, this decline in sterol biosynthesis and accumulation was correlated with the reduced accumulation of oleanolic acid glycosides compensated by a higher level of saponin secretion. With growing concentration of supplied silver ions, the content of sterols progressively increased, exceeding the control level by 22% at 150 µM Ag^+^ (however, this concentration was highly detrimental to hairy root growth). Simultaneously, the ratio of the two most abundant sterols, stigmasterol and sitosterol, was remarkably changed, equaling 2.5:1 for the control and 0.9:1 after treatment with 50 μM Ag^+^, where sitosterol was the most abundant sterol instead of stigmasterol.

In contrast to the effect exerted by Ag^+^, all the applied concentrations of Cd^2+^ increased the sterol accumulation in the CC16 line hairy roots (Table 2). This effect was directly concentration-dependent, from a 19% increase at 25 μM Cd^2+^ up to a level of 150% of the control at 150 μM Cd^2+^. The amount of predominant stigmasterol was similar at all the tested Cd^2+^ concentrations (on average 30% higher than the control level), whereas the amount of sitosterol decreased significantly (to 50% of the control) at the lowest applied concentration of Cd^2+^. Thus, the ratio of the stigmasterol and sitosterol was again substantially changed, equaling 6.6:1 at 25 μM Cd^2+^ and 2.2:1 at 150 μM Cd^2+^.

After elicitation with silver ions, the content of sterols in the CH2 line decreased by approximately 30% compared to the control at all the applied concentrations of Ag^+^ (Table 3). The ratio of stigmasterol to sitosterol was changed, equaling 1.4:1 in the control and approximately 0.9:1 in all the Ag-treated hairy root samples, so sitosterol became the predominant sterol instead of stigmasterol.

In contrast to the influence of cadmium exerted on the CC16 line, where the content of sterols was elevated in comparison to the control, with the CH2 line a decline in sterol accumulation was observed, with the lowest level (a 33% decrease compared to the control) at the concentration of 25 μM Cd^2+^ (Table 4). Similarly as described above, sitosterol became the prevailing compound, with stigmasterol: sitosterol ratios of 0.8:1 at 25 and 50 μM Cd^2+^, and 0.6:1 at 100 μM Cd^2+^.

Various applications of heavy metals as abiotic elicitors enhancing the productivity of in vitro cultures have been reported so far. For example, silver and cadmium ions at a concentration of 25 μM increased considerably (more than 11-fold) the content of diterpene quinines (referred to as tashinones) in *Salvia miltiorrhiza* cell suspension cultures [20]. Silver nitrate at a concentration of 1mM increased by 5- and 8-fold the accumulation of scopolamine and hyoscyamine, respectively, in hairy root cultures of *Brugmansia candida*; moreover, it stimulated a 3-fold increase in the release of scopolamine into the medium [21]. However, the toxic influence of high concentrations of heavy metal ions cannot be neglected, since sometimes the inhibition of hairy root growth was observed, and it was associated with a decrease in the accumulation of metabolites and an increase in their release. The higher release of compounds to the medium could be due to damage to the cell membrane or to cellular lysis, appearing as a consequence of heavy metal toxicity [21].

Summarizing the results obtained in the present study, it has to be admitted that the influence of silver and cadmium ions exerted various concentration-dependent effects that were different in the two tested hairy root lines. Generally, the accumulation of oleanolic acid glycosides in the hairy root tissue of both lines was reduced, whereas the main phenomenon observed as a response to heavy metal treatment was the strong stimulation (up to 12-fold) of the secretion of oleanolic acid saponins. The influence of both supplied heavy metal ions on sterol biosynthesis was particularly specific. In the CC16 line, the total content of sterols increased (up to 22%) for all the applied concentrations of Cd^2+^ and the two highest (100 and 150 μM) concentrations of Ag^+^. Simultaneously, the ratio of stigmasterol to sitosterol was significantly changed, with the growing amount of sitosterol finally exceeding the predominant stigmasterol. In the CH2 line, the total content of sterols declined for all applied Ag^+^ and Cd^2+^ concentrations; however, the phenomenon of the increased biosynthesis of sitosterol and its prevalence over stigmasterol was also observed. Thus, heavy metals seem to deeply disturb the triterpenoid biosynthesis pathway, since their influence includes modifications concerning not only the switch between sterols and pentacyclic compounds, but also significant changes in the sterol profile. Nevertheless, the advantage of the use of heavy metals as elicitors is debatable due to their harmful influence on hairy root growth and the visible changes in the roots’ morphology and branching.

#### 2.2.2. Effect of Ultrasound

Elicitation of hairy roots with ultrasound was performed according to the procedure described in chapter 3.2.2. After exposure to ultrasound, the hairy root samples were cultured during the subsequent 7 days. The fresh weight of both lines of hairy roots exposed to a 10 min treatment with ultrasound increased by 11% and 8% for the CC16 line and CH2 line, respectively. After 20 min of exposure, the fresh weight of the CC16 line roots remained the same as the control, whereas the fresh weight of the CH2 line roots decreased by 40% (Figure S4).

The dry weight increased by approximately 7% for the CH2 root line exposed to 10 min of ultrasound treatment, and it remained practically unchanged for the CC16 line. The dry weight of hairy roots exposed to 20 min ultrasound elicitation decreased, particularly remarkably (by 47%) for the CH2 line (Figure S5). Ultrasound treatment changed the color of the roots to a darker shade for both the CC16 and CH2 lines, particularly in samples after 20 min exposure (Figure S6).

**Figure 6:**
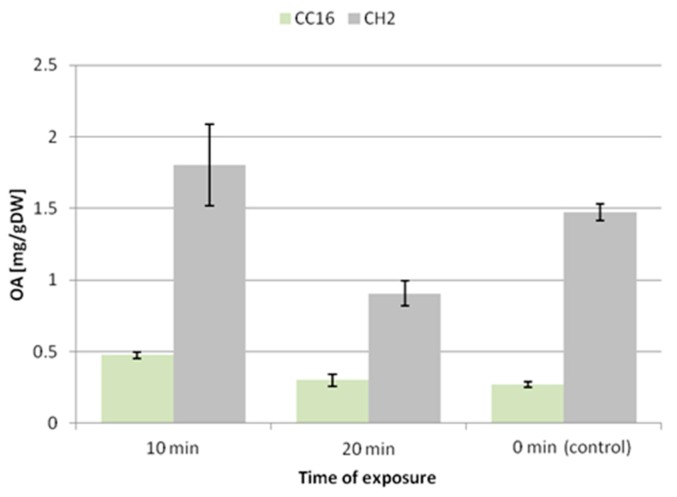
The content of oleanolic acid in hairy root tissue after elicitation with ultrasound. OA—oleanolic acid.

The 10 min exposure to ultrasound caused an increase in the accumulation of oleanolic acid glycosides by 50% and 64% in the hairy root tissue of the CC16 and CH2 lines, respectively. In turn, the 20 min exposure did not exert a significant effect on the CC16 line, whereas it decreased the level of saponins by 26% in the CH2 line (Figure 6).

**Figure 7:**
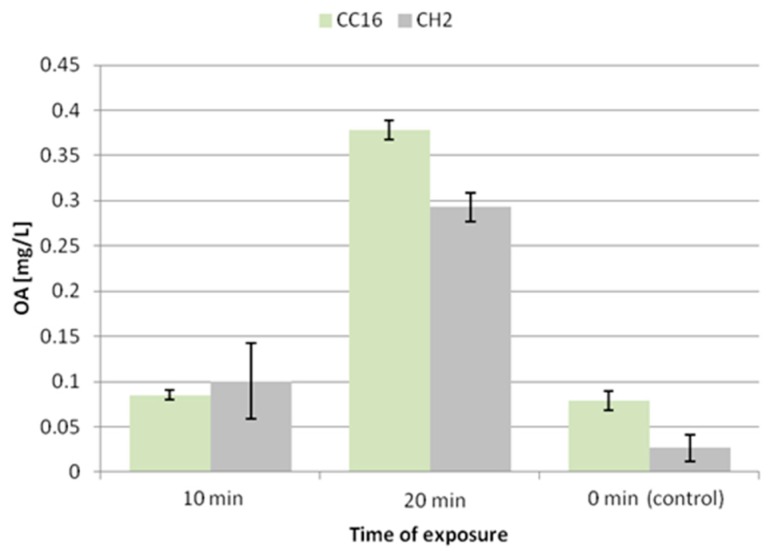
The content of oleanolic acid in the medium after elicitation with ultrasound. OA—oleanolic acid.

After 10 min exposure to ultrasound, the secretion of oleanolic acid glycosides did not remarkably change in the hairy root CC16 line, while it increased 3.8-fold in the CH2 line (Figure 7). A significant enhancement of the secretion was noted after 20 min exposure, 4-fold in the CC16 line and 11-fold in CH2 line. Thus, the elevated secretion of saponins after 20 min exposure to ultrasound is correlated with the reduction of the accumulation of these compounds in hairy root tissue.

The content of sterols increased in the hairy roots of both lines exposed to 10 min of ultrasound treatment, by 7% and 11% in the CC16 and CH2 lines, respectively, and it significantly decreased by 38% and 47% after 20 min of ultrasound treatment, without changing the ratio among individual compounds (Table 5 and Table 6).

Ultrasound has been often applied as a physical elicitor for stimulation and manipulation of cells, tissues, and organs in various plant in vitro techniques. It was reported that low-energy ultrasound enhanced the synthesis and secretion of shikonin to the culture media from a suspension culture of *Lithospermum erythrorhizon* [22]. Therefore, ultrasound was regarded as working in two ways: it increased the membrane permeability—shikonin was excreted to the medium 3.5-times more effectively, and it stimulated the activity of key enzymes responsible for shikonin synthesis, i.e., phenylalanine ammonia lyase and *p*-hydroxybenzoic acid geranyltransferase. The selection of appropriate parameters is crucial for reducing the level of mechanical damage and for permitting the reversible solubilization of various cellular membranes.

Nevertheless, ultrasound does not always improve the exudation of metabolites. Safari et al. (2012) reported a remarkable enhancement in the production of taxanes in hazel (*Corylus avellana*) suspension culture elicited by ultrasound, with no parallel increase in their release through the cell membrane [23]. Likewise, the production of cichoric, caftaric, chlorogenic, and caffeic acid was enhanced in ultrasound-elicitated *Echinacea purpurea* hairy root culture; however, these compounds were not detected in the liquid medium [24].

The present study revealed that, in the case of *C. officinalis* hairy root cultures, the effect of ultrasound of 50 kHz frequency markedly depended on the time of exposure. A short-time exposure caused an enhancement in the accumulation of oleanolic acid glycosides in the root tissues (50%–64%), accompanied by a slight increase in sterol content (7%–12%), whereas the secretion of saponins did not change in the CC16 line and increased almost 4-fold in the CH2 line. A longer exposure caused a decline in the level of saponins only in the tissue of the hairy root CH2 line, while in the less sensitive CC16 line it remained comparable to the control. 20 min of ultrasound treatment significantly stimulated the secretion of oleanolic acid saponins (4-fold in the CC16 line and 11-fold in the CH2 line), simultaneously decreasing the content of sterols (by 38%–46%). No changes in the ratio among individual sterols were noted.

#### 2.2.3. Effect of UV radiation

Elicitation of hairy roots with UV-C radiation was performed according to the procedure described in chapter 3.2.3. After every exposure to UV radiation, the hairy root samples were cultured during the subsequent 7 days. Thirty minutes of UV exposure resulted in a slight decrease in the fresh weight of both hairy root lines, by 13 % for CC16 and 15 % for CH2. After 60 min UV exposure, a decline in fresh weight (by 14%) was noted only for the CH2 line (Figure S7). The effect of UV radiation on the hairy root dry weight was also not very prominent. After 30 min exposure it decreased by 13% and 8% in the CC16 and CH2 hairy root lines, respectively. After 60 min UV exposure, again a decline in dry weight (by 15%) was noted only for the CH2 line (Figure S8). Neither the root color nor their morphology and branching were significantly changed.

**Figure 8:**
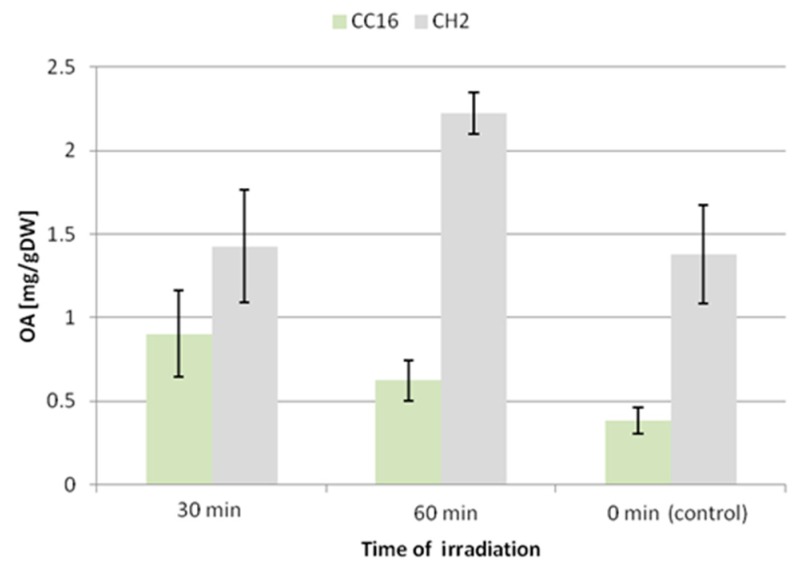
The content of oleanolic acid in hairy root tissue after elicitation with UV radiation. OA—oleanolic acid.

The accumulation of oleanolic acid glycosides was elevated in both hairy root lines; however, the CC16 hairy roots seemed to be more susceptible to this elicitation. After 30 and 60 min of UV exposure the accumulation of saponins in this line increased 2.4-fold and 1.6-fold, respectively. In turn, the content of saponins in CH2 hairy roots practically did not change in samples subjected to 30 min UV exposure (increased by 3%, statistically not significant), and it increased by 38% after 60 min exposure (Figure 8).

**Figure 9:**
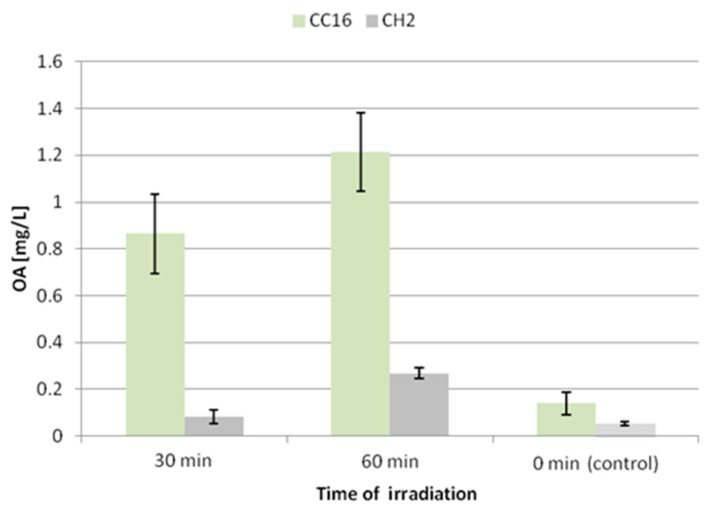
The content of oleanolic acid in the medium after elicitation with UV radiation. OA—oleanolic acid.

A similar effect was noted for the secretion of oleanolic acid glycosides (Figure 9). The concentration of oleanolic acid in the medium for the CC16 line increased 6-fold and 8.5-fold after 30 and 60 min of UV exposure, respectively. This enhancement of the secretion of saponins was less significant (1.5-fold and 5-fold after 30 and 60 min of UV exposure, respectively) in the CH2 line.

In both hairy root lines the content of sterols increased, in samples exposed to 30 min UV radiation by 6% and 13% in the CC16 and CH2 lines, respectively, and by 15% and 27% in samples after 60 min of exposure (Table 7 and Table 8). However, the increase noted for the CC16 line was not statistically significant. Again, as after ultrasound treatment, no remarkable changes in the ratio among individual compounds were noted.

Application of UV light has often been reported to influence the production of compounds protective against this potentially harmful irradiation, such as anthocyanins [25]. However, both UV-B (280–315 nm) and UV-C (below 280 nm) were reported to exert stimulatory effects on the production of other metabolites, e.g., UV-B stimulated the synthesis of indole alkaloids in *Catharanthus roseus* hairy root cultures, while UV-C promoted the synthesis of resveratrol and other stilbenes in callus and cell suspension cultures of various *Vitis vinifera* genotypes [9]. Nowadays, there is also an increasing interest in applying UV-B or UV-C radiation to enhance the concentration of health-promoting secondary plant metabolites in plant-based foods, especially during postharvest and storage. Such treatment can lead to enhanced accumulation of flavonoids or carotenoids [26]; however, according to the results obtained in our study, its influence on the triterpenoid content of plant material also cannot be ruled out.

Thus, the two *C. officinalis* hairy root lines displayed different susceptibilities to UV-C radiation. In the CC16 line, the content of oleanolic acid saponins accumulated in hairy root tissue was enhanced 2.4-fold and 1.6-fold after 30 and 60 min of UV exposure, respectively, whereas the saponin content increased by only 38% after 60 min exposure in the CH2 line. Saponin secretion increased 6-fold and 8.5-fold after 30 and 60 min of UV treatment in the CC16 line, and 1.5-fold and 5-fold in the CH2 line. In turn, the content of sterols increased more significantly in the CH2 line (by 13% and 27% after 30 and 60 min of treatment, respectively). As in the case of ultrasound, again no changes in the ratio among individual sterols were observed.

## 3. Material and Methods

### 3.1. Plant Material

Hairy roots were obtained after transformation with wild-type *Agrobacterium rhizogenes* strain ATCC 15834 and maintained as described by Długosz et al. (2013) [14]. The CC16 line was derived from cotyledon and the CH2 line was from hypocotyl explants [13]. The obtained hairy root cultures were subcultured every 3–4 weeks by the transfer of 1–2 pieces cut off from the young branched root to 50 mL Erlenmeyer flasks with 25 mL of fresh medium; these were kept in darkness at 23 ± 2 °C and shaken at 120 rpm.

### 3.2. Elicitation of Hairy Root Cultures

#### 3.2.1. Elicitation with Ag^+^ and Cd^2+^ Ions

Silver ion Ag^+^ was supplied as silver nitrate (AgNO_3_, Sigma S7276, Poznań, Poland). Cadmium ion Cd^2+^ was supplied as cadmium chloride (CdCl_2_, Sigma 202908). Appropriate weighed samples of powder were dissolved in deionized water to obtain a concentration of 50 mM. Solutions were sterilized by filtration through a 0.22 μm syringe filter (Millipore, Bionovo, Legnica, Poland) and added to the culture medium to obtain final concentrations of 25, 50, 100, and 150 μM.

Small portions (i.e., inoculum consisting of single intact fragment of branched root) of fresh hairy roots derived from 3-week liquid culture of the two lines (CC16 and CH2) were placed separately in 300 mL Erlenmeyer flasks containing 50 mL of medium (½MS). After 7 days, the medium was topped up to 100 mL of ½MS and simultaneously the samples of both lines were supplied with elicitor. The CC16 line was treated with four concentrations of silver nitrate and cadmium chloride: 25, 50, 100, and 150 μM; however, the highest concentration (150 μM) of both salts exhibited visibly harmful effects on the hairy roots. Therefore, the CH2 line was treated only with concentrations of 25, 50, and 100 μM. As previously, incubation lasted 7 days.

#### 3.2.2. Elicitation with Ultrasound

An ultrasonic bath (Polsonic Model IP32 D, Warsaw, Poland) was used to elicitate the hairy root cultures. The bath had a fixed frequency of 50 kHz with an average power of 250 W. Two periods of ultrasound exposure, 10 and 20 min, were applied for the elicitation of samples of hairy roots of the tested CC16 and CH2 lines, prepared as described previously. After ultrasound treatment, the tested samples and untreated controls (in 3 replicates each) were incubated in typical conditions (darkness, 24 °C, rotation at 120 rpm) during the subsequent 7 days.

#### 3.2.3. Elicitation with Ultraviolet (UV) Radiation

Hairy root cultures were exposed to UV-C radiation during two periods, 30 and 60 min. Samples of both lines (CC16 and CH2) were placed in a laminar cabinet Alpina (Konin, Poland) at a distance of 40 cm from a UV-C tube (LSE Lighting, Worcester, UK) 15 W, 90 μW/cm) emitting radiation at λ =254 nm. The flasks were kept open during irradiation. Subsequently, they were placed on a rotary shaker at 120 rpm in darkness and incubated for the following 7 days.

### 3.3. Extraction and Fractionation

#### 3.3.1. Extraction of Hairy Roots

Hairy roots were filtered from the medium using a Büchner funnel under vacuum. The hairy roots were weighed immediately and, after drying at room temperature, they were powdered using a grinding mortar. The powders were extracted, first with diethyl ether, then methanol, using a Soxhlet extractor over 8 h. The extracts were evaporated to dryness at 40 °C under reduced pressure in a rotary evaporator.

#### 3.3.2. Extraction of Culture Medium

The measured volume of the liquid medium filtered from the hairy roots was extracted 3-times with 20 mL portions of *n*-butanol. The organic layers were combined and washed with *n*-butanol saturated water (once with the same volume as the volume of the extract). The obtained butanolic extract was evaporated to dryness in a rotary evaporator.

#### 3.3.3. Fractionation of Hairy Root Diethyl Ether Extracts

Evaporated diethyl ether extracts from the hairy roots were fractionated by adsorption preparative TLC on 20 cm × 20 cm glass plates coated manually with silica gel 60H (Merck, Darmstadt, Germany). The solvent system chloroform: methanol 97:3 (*v/v*) was applied for developing. Three fractions were obtained as described by Szakiel et al. (2012): free (non-esterified) triterpenoids, triterpene acids, and triterpenoid esters [27]. Subsequently, fractions containing free neutral triterpenes and sterols (*R*_F_ 0.3–0.9) were directly analyzed by GC-MS, fractions containing triterpene acids (*R*_F_ 0.2–0.3) were methylated with diazomethane, and fractions containing triterpenoid esters (*R*_F_ 0.9–1) were subjected to alkaline hydrolysis.

### 3.4. Quantification of Oleanolic Acid by GC

The samples were dissolved in suitable portions of a diethyl ether: methanol 3:1 (*v/v*) mixture. Quantitative measurement of oleanolic acid (in the form of its methyl ester) was performed by gas-liquid chromatography (GLC) at 270 °C on a Shimadzu GC-2014 instrument equipped with a flame ionization detector. Samples were applied by split injection 1:5 on a ZB-1 30 m × 0.25 mm × 0.25 μm column (Phenomenex, SHIM-POL, Izabelin, Poland). The temperature of the injector and detector was 290 °C. Nitrogen was used as the carrier gas at a flow rate of 1.2 mL/min. Peak identification and quantification of oleanolic acid were carried out by referring to a calibration curve prepared with an authenticated sample of methylated oleanolic acid as the standard [28].

### 3.5. Identification and Quantification of Triterpenoids by GC-MS/FID

An Agilent Technologies 7890A gas chromatograph (Perlan Technologies, Warszawa, Poland) equipped with a 5975C mass selective detector was used for qualitative and quantitative analyses. Samples dissolved in diethyl ether:methanol (5:1, *v/v*) were applied (in a volume of 1–4 μL) using 1:10 split injection. The column used was a 30 m × 0.25 mm i.d., 0.25-μm, HP-5MS UI (Agilent Technologies, Santa Clara, CA, USA). Helium was used as the carrier gas at a flow rate of 1 mL/min. The separation was made either under isothermal conditions at 280 °C or with the following temperature program: initial temperature of 160 °C held for 2 min, then increased to 280 °C at 5 °C/min, and the final temperature of 280 °C held for a further 44 min. The other employed parameters were as follows: inlet and FID (flame ionization detector) temperature 290 °C; MS transfer line temperature 275 °C; quadrupole temperature 150 °C; ion source temperature 230 °C; EI 70 eV; *m/z* range 33–500; FID gas (H_2_) flow 30 mL·min^−1^ (hydrogen generator HydroGen PH300, Peak Scientific, Inchinnan, UK); and air flow 400 mL·min^−1^. Individual compounds were identified by comparing their mass spectra with library data from Wiley 9^th^ ED. and NIST 2008 Lib. SW Version 2010, or previously reported data, and by comparison of their retention times and corresponding mass spectra with those of authentic standards, where available. Quantitation of steroids was performed using an external standard method based on calibration curves determined for an authentic standard of sitosterol [29].

### 3.6. Statistical Analysis of Data

All experiments were performed in triplicate. Data are presented as the means ± standard deviation of three independent samples analyzed in triplicate. The data were subjected to one-way analysis of variance (ANOVA), and the differences between means were evaluated using Duncan’s multiple-range test. Statistical significance was considered to be obtained at *p* < 0.05.

## 4. Conclusions

Despite the numerous studies performed with the use of different experimental models, the mechanism of action of the majority of elicitors applied in plant in vitro cultures has not been sufficiently elucidated. Therefore the trials of productivity enhancement of in vitro cultures are still rather arbitrary and they require case-by-case experiments. In this study selected abiotic elicitors were applied, and their efficiency in the stimulation of triterpenoid biosynthesis was evaluated. The two *C. officinalis* hairy root lines tested in the present work differ in tissue origin: one line was derived from cotyledon and the other from hypocotyl explants. Their sensibility to various elicitors as well as the mechanism of their response to stresses might be different, and such phenomenon is often observed in plant in vitro cultures [13,15]. Some of these differences can be at least partially explained as dependent on the origin of the explants, particularly in case of the treatment with silver and cadmium ions, since the tolerance to toxicity of heavy metals and the mechanisms of their avoidance or accumulation are tissue-specific in plants [30].

Trials of the stimulation of triterpenoid biosynthesis in plants and plant in vitro cultures often concern the possible competition of pathways leading to sterols and other triterpenoids (mainly pentacyclic). Since both sterols and pentacyclic triterpenoids are synthesized as products of 2,3-oxidosqualene cyclization, it can be expected that biosynthesis of triterpenoids occurs when sterol formation is already satisfied or, in urgent situations, sacrificed. However, the simultaneous enhancement of these two groups of compounds has also been observed, for example, in the experimental enhancement of triterpenoid production in *Panax ginseng* [31]. As previously reported [13] the symptoms of the competition between the biosynthetic pathways of sterols and pentacyclic triterpenoids were the most visible in the reaction of *C. officinalis* hairy roots to stimulation with the biotic elicitors jasmonic acid and chitosan. In the present study on abiotic stressors, this phenomenon is much less evident, particularly regarding UV irradiation, which caused an increase in the content of both investigated groups of triterpenoids. Therefore, regarding the influence of elicitation on the biosynthetic pathways of sterols and pentacyclic triterpenoids, it can be concluded that the mechanism of their regulation does not consist only in the supposed redirection of the carbon flow at the branch point, or limitations on substrate availability.

## Figures and Tables

**Table 1:** The content of sterols in line CC16 hairy root tissue after treatment with Ag^+^ ions.

Compound	Control	Ag^+^ Concentration [μM]			
		25	50	100	150
	μg/g D.W.				
cholesterol	17.65 ± 1.93a	21.87 ± 2.64a	18.25 ± 1.88a	25.30 ± 2.40a	41.66 ± 3.49b
campesterol	30.79 ± 2.27a	14.86 ± 2.00b	20.47 ± 1.51c	23.44 ± 2.42c	34.72 ± 1.88a
stigmasterol	275.95 ± 23.19a	133.82 ± 11.36b	95.23 ± 9.89c	197.23 ± 20.32d	216.32 ± 20.39d
sitosterol	110.56 ± 23.28a	86.21 ± 8.47a	108.61 ± 9.05a	175.32 ± 10.70b	156.96 ± 13.17b
sitostanol	16.08 ± 7.95a	78.85 ± 3.77b	56.18 ± 5.34c	8.58 ± 6.84d	63.05 ± 6.49c
isofucosterol	9.20 ± 2.28a	n.d.	5.91 ± 0.60b	n.d.	20.06 ± 2.69c
Total	460.23	285.61	304.65	429.87	562.71

Results are referenced to hairy root dry mass and expressed as mean ± SD of three independent samples. Results not sharing a common letter are significantly different (*p* < 0.05). n.d.—not detected.

**Table 2:** The content of sterols in line CC16 hairy root tissue after treatment with Cd^2+^ ions.

Compound	Control	Cd^2+^ Concentration [μM]			
		25	50	100	150
	μg/g D.W.				
cholesterol	17.65 ± 1.93a	35.07 ± 2.78b	30.09 ± 2.52b	49.78 ± 2.53c	55.47 ± 5.47c
campesterol	30.79 ± 2.27a	32.79 ± 1.92a	35.65 ± 2.01a	19.22 ± 1.70b	27.06 ± 2.98c
stigmasterol	275.95 ± 23.19a	363.62 ± 35.88b	345.13 ± 40.68b	333.24 ± 31.95b	346.18 ± 40.66b
sitosterol	110.56 ± 23.28a	55.69 ± 5.19b	103.68 ± 10.71a	111.74 ± 10.25a	154.80 ± 16.91c
sitostanol	16.08 ± 7.95a	44.12 ± 3.13b	76.22 ± 8.07c	70.71 ± 4.86c	73.17 ± 5.30c
isofucosterol	9.20 ± 2.28a	15.69 ± 0.80b	22.25 ± 2.33c	41.19 ± 3.75d	28.76 ± 3.26e
Total	460.23	546.98	613.02	625.88	685.44

Results are referenced to hairy root dry mass and expressed as mean ± SD of three independent samples. Results not sharing a common letter are significantly different (*p* < 0.05). n.d.—not detected.

**Table 3:** The content of sterols in line CH2 hairy root tissue after treatment with Ag^+^ ions.

Compound	Control	Ag^+^ Concentration [μM]		
		25	50	100
	μg/g D.W.			
cholesterol	10.83 ± 1.95a	9.93 ± 1.07a	9.09 ± 1.10a	5.69 ± 0.73b
campesterol	29.59 ± 3.57a	13.22 ± 1.54b	15.69 ± 1.47b	7.09 ± 0.89c
stigmasterol	226.24 ± 32.86a	139.77 ± 15.03b	127.98 ± 13.02b	132.57 ± 15.27b
sitosterol	165.26 ± 20.04a	152.25 ± 16.40a	133.09 ± 15.11a	146.02 ± 15.68a
sitostanol	18.24 ± 2.16a	17.72 ± 2.08a	15.11 ± 1.63a	15.42 ± 1.42a
isofucosterol	26.37 ± 3.21a	25.81 ± 2.67a	33.4 ± 3.08b	22.40 ± 2.44a
Total	476.53	358.7	334.18	329.19

Results are referenced to hairy root dry mass and expressed as mean ± SD of three independent samples. Results not sharing a common letter are significantly different (*p* < 0.05).

**Table 4:** The content of sterols in line CH2 hairy root tissue after treatment with Cd^2+^ ions.

Compound	Control	Cd^2+^ Concentration [μM]		
		25	50	100
	μg/g D.W.			
cholesterol	10.83 ± 1.57a	15.4 ± 1.82b	16.63 ± 1.85b	23.18 ± 2.42c
campesterol	29.59 ± 3.03a	12.59 ± 1.50b	21.61 ± 2.29c	61.75 ± 6.59d
stigmasterol	226.24 ± 25.68a	126.06 ± 15.54b	143.97 ± 16.01b	119.64 ± 13.04b
sitosterol	165.26 ± 15.04a	163.13 ± 16.97a	183.08 ± 20.12a	191.93 ± 20.25a
sitostanol	18.24 ± 2.62a	13.76 ± 1.44b	15.81 ± 1.70b	21.68 ± 2.32a
isofucosterol	26.37 ± 3.11a	36.73 ± 4.03b	38.38 ± 4.06b	48.43 ± 5.15c
Total	476.53	367.6	419.48	466.60

Results are referenced to hairy root dry mass and expressed as mean ± SD of three independent samples. Results not sharing a common letter are significantly different (*p* < 0.05).

**Table 5:** The content of sterols in line CC16 hairy root tissue after elicitation with ultrasound.

Compound	Control	Time of Exposure to Ultrasound [min]	
		10	20
	μg/g D.W.		
cholesterol	20.23 ± 2.95a	10.88 ± 1.24b	4.38 ± 0.52c
campesterol	25.90 ± 3.01a	16.41 ± 1.93b	11.92 ± 1.30b
stigmasterol	227.81 ± 31.27a	301.07 ± 32.15b	180.73 ± 19.55c
sitosterol	102.08 ± 11.92a	86.65 ± 9.40a	42.88 ± 5.24b
sitostanol	9.20 ± 1.45a	7.05 ± 0.81a,b	5.13 ± 1.21b
isofucosterol	10.28 ± 1.26a	n.d.	n.d.
Total	395.50	422.06	245.04

Results are referenced to hairy root dry mass and expressed as mean ± SD of three independent samples. Results not sharing a common letter are significantly different (*p* < 0.05). n.d.—not detected.

**Table 6:** The content of sterols in line CH2 hairy root tissue after elicitation with ultrasound.

Compound	Control	Time of Exposure to Ultrasound [min]	
		10	20
	μg/g D.W.		
cholesterol	10.38 ± 1.22a	10.04 ± 1.14a	8.36 ± 1.60a
campesterol	28.55 ± 3.11a	31.91 ± 3.05a	15.91 ± 2.63b
stigmasterol	242.37 ± 25.15a	264.77 ± 28.31a	122.65 ± 25.92b
sitosterol	105.94 ± 11.08a	128.61 ± 13.09a	59.97 ± 18.13b
sitostanol	10.25 ± 1.20a	13.62 ± 1.54a	9.45 ± 0.99a
isofucosterol	21.67 ± 2.33a	23.47 ± 2.53a	8.62 ± 2.82b
Total	419.16	472.42	224.96

Results are referenced to hairy root dry mass and expressed as mean ± SD of three independent samples. Results not sharing a common letter are significantly different (*p* < 0.05).

**Table 7:** The content of sterols in line CC16 hairy root tissue after elicitation with UV radiation.

Compound	Control	Time of Exposure to Ultrasound [min]	
		30	60
	μg/g D.W.		
cholesterol	19.55 ± 2.20a	18.78 ± 2.04a	22.85 ± 2.40a
campesterol	21.64 ± 2.38a	21.43 ± 2.17a	25.75 ± 3.01a
stigmasterol	225.54 ± 25.96a	250.88 ± 30.64a	276.26 ± 32.78a
sitosterol	99.89 ± 11.75a	100.96 ± 12.26a	113.02 ± 14.16a
sitostanol	9.20 ± 1.02a	9.65 ± 1.07a	9.91 ± 1.07a
isofucosterol	12.72 ± 1.44a	10.68 ± 1.92a	11.38 ± 1.52a
Total	388.54	412.38	459.17

Results are referenced to hairy root dry mass and expressed as mean ± SD of three independent samples. Results not sharing a common letter are significantly different (*p* < 0.05).

**Table 8:** The content of sterols in line CH2 hairy root tissue after elicitation with UV radiation.

Compound	Control	Time of Exposure to Ultrasound [min]	
		30	60
	μg/g D.W.		
cholesterol	11.36 ± 1.84a	12.43 ± 1.41a	12.80 ± 1.42a
campesterol	24.21 ± 2.67a	29.04 ± 3.62a	37.64 ± 4.18b
stigmasterol	239.23 ± 26.50a	276.38 ± 30.04a,b	314.71 ± 33.57b
sitosterol	108.39 ± 12.03a	124.07 ± 14.15a,b	155.75 ± 20.09b
sitostanol	12.09 ± 1.35a	13.01 ± 1.57a	18.50 ± 2.22b
isofucosterol	17.94 ± 2.02a	19.06 ± 2.18a	22.62 ± 3.06a
Total	413.22	473.99	562.02

Results are referenced to hairy root dry mass and expressed as mean ± SD of three independent samples. Results not sharing a common letter are significantly different (*p* < 0.05).

## Supplementary Materials

The following are available online at https://www.mdpi.com/1420-3049/24/16/2907/s1, Figure S1: Fresh weight of the line CC16 hairy roots after 7 days of elicitation with silver nitrate or cadmium chloride (25, 50, 100, 150 µM) compared to the control sample. Figure S2: Dry weight of the line CC16 hairy roots after 7 days of elicitation with silver nitrate or cadmium chloride (25, 50, 100, 150 µM) compared to the control sample. Figure S3: Hairy root morphology (line CH2) after 7 days of treatment with 100 µM Ag^+^ and the control culture. Figure S4: Fresh weight of the CC16 and CH2 hairy root lines after elicitation with ultrasound. Figure S5: Dry weight of the CC16 and CH2 hairy root lines after elicitation with ultrasound. Figure S6: Hairy root morphology (line CH2) after 10 min and 20 min of exposure to ultrasound and 7 days of subsequent culture, compared to the control. Figure S7: Fresh weight of the CC16 and CH2 hairy root lines after elicitation with UV-C radiation. Figure S8: Dry weight of the CC16 and CH2 hairy root lines after elicitation with UV-C radiation.
