# Supplementary material for: Influence of Selected Abiotic Factors on Triterpenoid Biosynthesis and Saponin Secretion in Marigold (Calendula officinalis L.) in Vitro Hairy Root Cultures
Source: Molecules. 2019 Aug 10;24(16):2907. doi: 10.3390/molecules24162907 (PMC6720177; doi:10.3390/molecules24162907)
Supplement: Supplementary file 1 [file molecules-24-02907-s001.pdf]

Influence of Selected Abiotic Factors on Triterpenoid Biosynthesis and Saponin Secretion in Marigold (Calendula officinalis L.) in Vitro Hairy Root Cultures

The line CC16 was treated by four concentrations of silver nitrate and cadmium chloride: 25, 50, 100 and 150 µM. All concentrations significantly reduced both the fresh mass and dry weight of the roots (by 47-77%, Fig. S1 and S2). The highest concentration (150 µM) of both salts exerted the visibly harmful effect on hairy roots, which became very dark and stopped to grow. Therefore, the line CH2 was treated only by concentrations of 25, 50 and 100 µM.

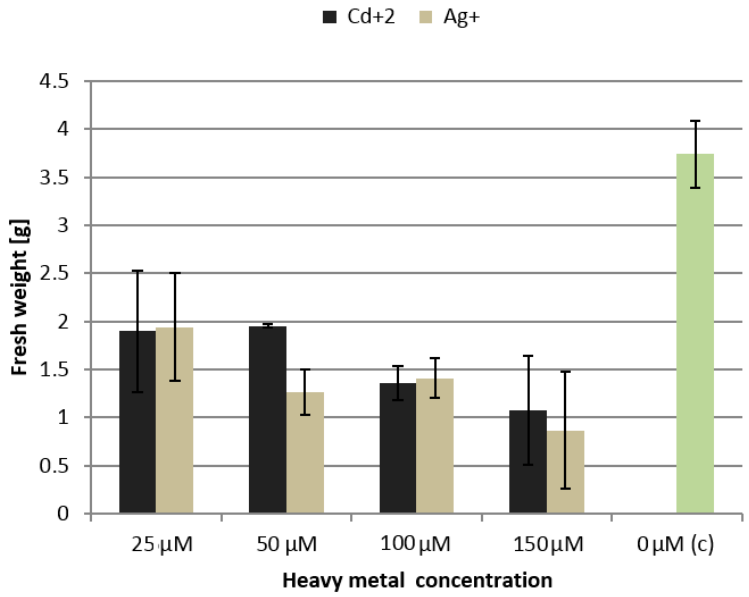

Figure S1. Fresh weight of the line CC16 hairy roots after 7 days of elicitation with silver nitrate or cadmium chloride (25, 50, 100, 150 µM) compared to the control sample.

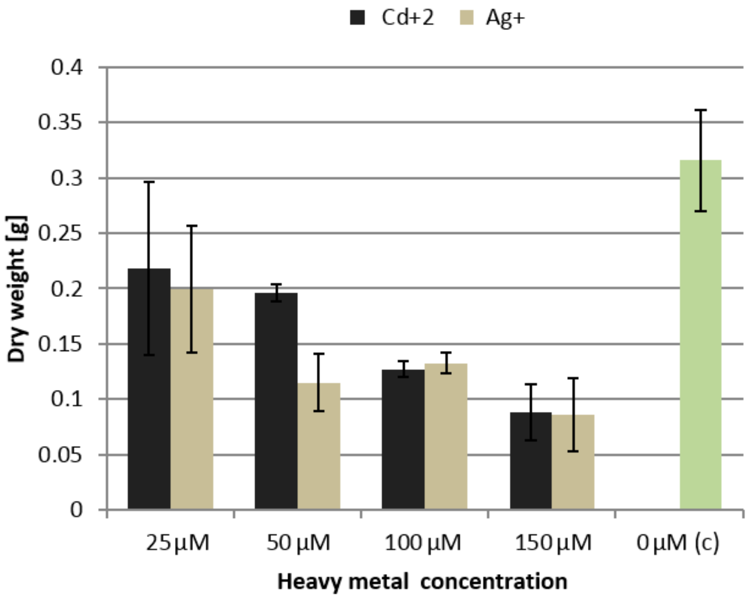

Figure S2. Dry weight of the line CC16 hairy roots after 7 days of elicitation with silver nitrate or cadmium chloride (25, 50, 100, 150 µM) compared to the control sample.

Elicitation with heavy metal ions led to significant changes in the root morphology. The roots became thicker and significantly less branched, particularly after Ag^+ treatment (Fig. 3S).

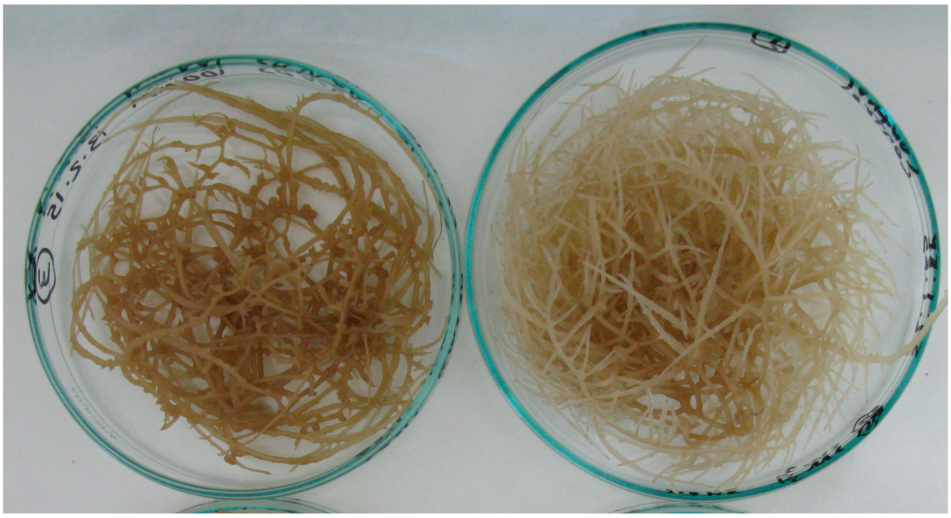

Figure S3. Hairy root morphology (line CH2) after 7 days of treatment with 100 µM Ag^+ (left) and the control culture (right).

Effect of ultrasound on hairy root growth

After the exposition to ultrasound, the hairy root samples were cultured during subsequent 7 days. The fresh weight of both lines of hairy roots exposed for 10 min treatment with ultrasound increased by 11% and 8% for CC16 line and CH2 line, respectively. After 20 min of the exposition, the fresh weight of the CC16 line roots remained the same as the control, whereas the fresh weight of the CH2 line roots decreased by 40% (Fig.S4).

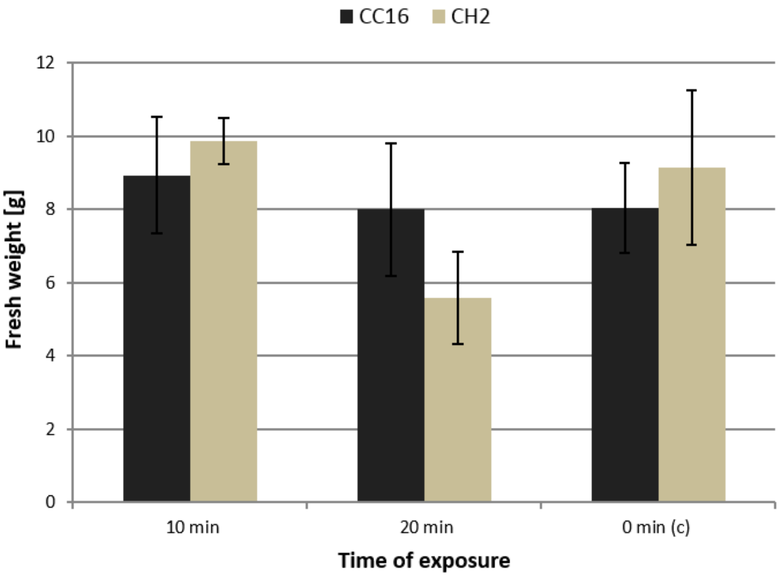

Figure S4. Fresh weight of the CC16 and CH2 hairy root lines after elicitation with ultrasound.

The dry weight increased approximately by 7% for CH2 line roots exposed to 10 min of ultrasound treatment, and it remained practically unchanged for CC16 line. The dry weight of hairy roots exposed to 20 min ultrasound elicitation decreased, particularly remarkably (by 47%) for the CH2 line (Fig. S5). Ultrasound treatment changed the color of the roots to darker for both lines CC16 and CH2, particularly in samples after 20 min exposure (Fig. S6).

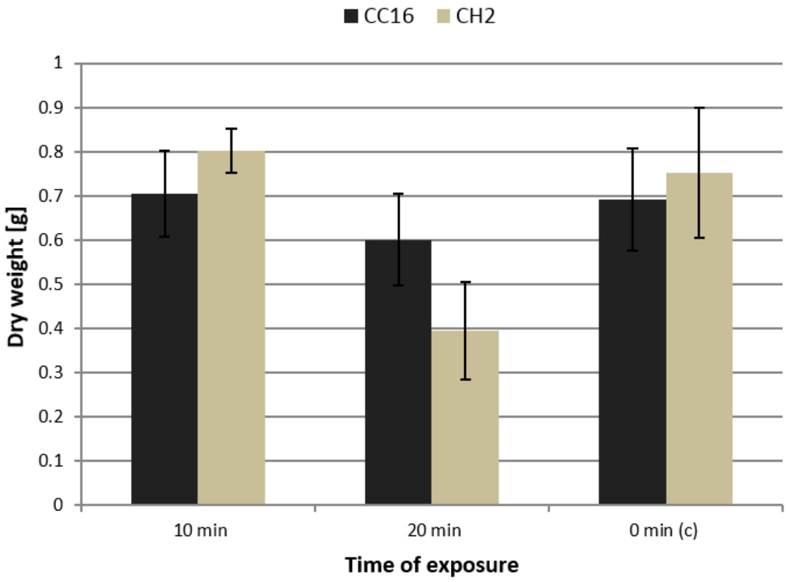

Figure 5S. Dry weight of the CC16 and CH2 hairy root lines after elicitation with ultrasound.

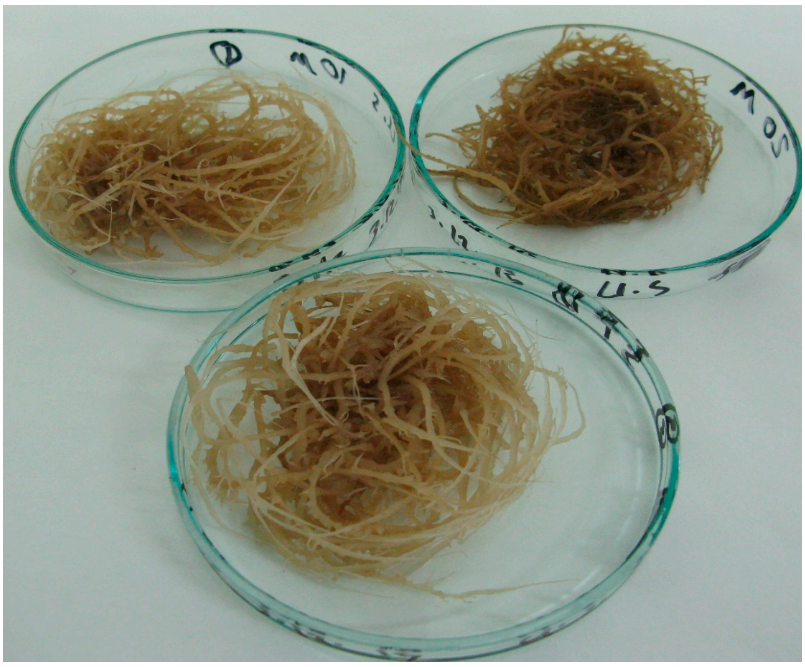

Figure S6. Hairy root morphology (line CH2) after 10 min (upper left) and 20 min (upper right) of exposure to ultrasound and 7 days of subsequent culture, compared to the control (central below).

Effect of UV radiation on hairy root growth

After every exposition to UV radiation, the hairy root samples were cultured during subsequent 7 days. 30 min of UV exposure resulted in a slight decrease of the fresh weight of both hairy root lines, by 13 % for CC16 and 15 % for CH2. After 60 min UV exposure the decline in fresh weight (by 14%) was noted only for the line CH2 (Fig. 7S). The effect of UV radiation on the hairy root dry weight was also not very prominent. After 30 min exposure it decreased by 13% and 8% in the CC16 and CH2 hairy root lines, respectively. After 60 min UV exposure, again the decline in dry weight (by 15%) was noted only for the line CH2 (Fig. 8S). Neither root color nor their morphology and branching were significantly changed.

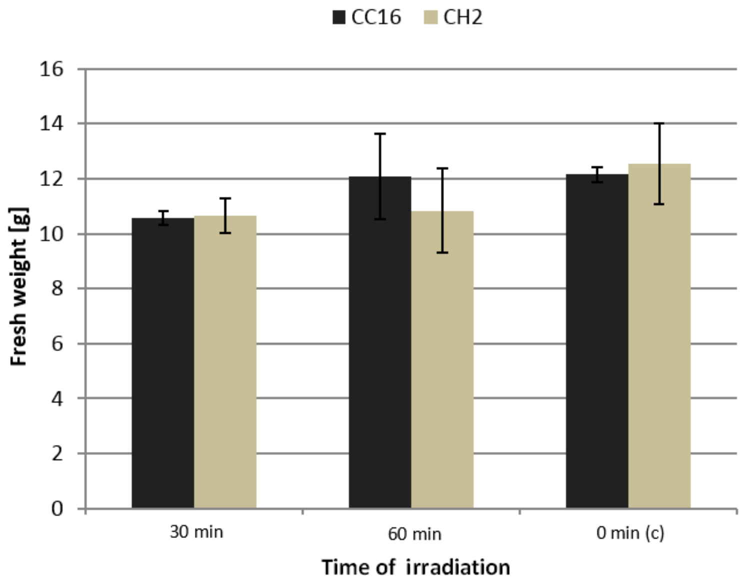

Figure S7. Fresh weight of the CC16 and CH2 hairy root lines after elicitation with UV-C radiation.

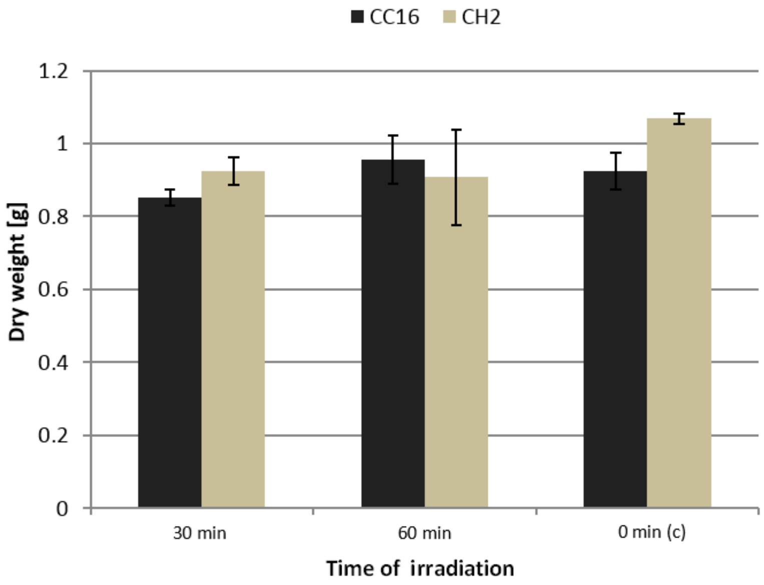

Figure S8. Dry weight of the CC16 and CH2 hairy root lines after elicitation with UV-C radiation.
